# Swedish perspectives and ethical discussion

**DOI:** 10.1192/j.eurpsy.2021.82

**Published:** 2021-08-13

**Authors:** D. Wasserman

**Affiliations:** National Centre For Suicide Research And Prevention Of Mental Lll-health, Karolinska Institutet, Stockholm, Sweden

**Keywords:** holistic approach, ethical questions, risk groups, Swedish COVID-19 response

## Abstract

As COVID-19 spread, many countries in the world responded swiftly in an attempt to reduce transmission. Sweden, however, took a different approach to many other nations and did not implement a nationwide lockdown, instead deciding on a more “holistic approach to public health”. The focus was on minimising transmission as much as possible, protecting those in risk groups, ensuring that the response strategies were sustainable long-term, mitigating other health concerns as a result of the response and that evidence-based methods were used as much as possible. At this stage, it is difficult to know how exactly the Swedish strategy has fared in comparison to other responses. In Sweden, there has been much debate about the strategy, particularly concerning the protection of the elderly due to unexpectedly high mortality rates in the older population as well as among residents in retirement homes. Many ethical questions remain in regard to which strategies would have been preferable.

**Disclosure:**

No significant relationships.

